# Increased prevalence of depression and anxiety in patients with migraine and interictal photophobia

**DOI:** 10.1186/s10194-016-0629-6

**Published:** 2016-04-14

**Authors:** Stephanie M. Llop, Jonathan E. Frandsen, Kathleen B. Digre, Bradley J. Katz, Alison V. Crum, Chong Zhang, Judith E. A. Warner

**Affiliations:** Department of Ophthalmology, University of Puerto Rico School of Medicine, PO Box 365067, San Juan, Puerto Rico 00936 USA; Department of Ophthalmology and Visual Sciences, John A. Moran Eye Center, University of Utah Health Sciences Center, 65 N. Mario Capecchi Drive, Salt Lake City, UT 84132 USA; Department of Neurology, University of Utah Health Sciences Center, 175 North Medical Drive East, Salt Lake City, UT 84132 USA; Division of Epidemiology, Department of Internal Medicine, University of Utah Health Sciences Center, 30 N 1900 East, Salt Lake City, UT 84132 USA

**Keywords:** Photophobia, Migraine, Depression, Anxiety, Sleep disorders, Intrinsically photosensitive retinal ganglion cells

## Abstract

**Background:**

Most patients with migraine report photophobia associated with headache; a subset report interictal photophobia. These patients are light sensitive even during headache-free periods. The objective of this case–control study was to assess the prevalence of symptoms of anxiety and depression in migraine patients with and without interictal photophobia.

**Methods:**

We recruited 16 subjects with migraine and interictal photophobia, 16 age- and gender-matched migraine subjects without interictal photophobia, and 16 age- and gender- matched controls. Migraine subjects met International Headache Society classification criteria. Participants completed a photophobia questionnaire, Beck Depression Inventory (BDI-II), and Beck Anxiety Inventory (BAI). Chi-square analyses and two-tailed Wilcoxon rank sum tests were used for the analyses.

**Results:**

Subjects with interictal photophobia had significantly higher scores on the photophobia questionnaire compared to subjects without interictal photophobia. Subjects with interictal photophobia had significantly higher scores on the BDI-II and BAI compared to subjects without interictal photophobia.

**Conclusions:**

Migraine patients with interictal photophobia are more likely to manifest symptoms of depression and anxiety compared to migraine patients without interictal photophobia. Care providers should be aware of increased prevalence of these symptoms in this population and consider appropriate referrals. Future research could assess whether treatment of photophobia leads to improvements in symptoms of depression and anxiety in migraine patients.

## Background

Photophobia is a general term used to describe light sensitivity or abnormal intolerance of light. Patients with photophobia avoid light because of pain or discomfort. In normal subjects, photophobia appears to serve the function of protecting the retina from damage due to photo-toxicity [1]. However, patients with an underlying disorder like migraine have been shown to display considerably greater light sensitivity when compared to controls [2]. Nearly all patients with migraine report photophobia during headache episodes, but a subset of these patients report photophobia all the time (“interictal photophobia”), even during headache–free periods [3]. Furthermore, interictal photophobia associated with migraine may have a negative, and sometimes disabling, impact on quality of life and social functioning, with the highest prevalence between the ages of 25 and 55 years, potentially the most productive period of life [4].

Several studies demonstrate that the presence of psychiatric co-morbidities is increased in people with migraine, especially depression and anxiety [5–10]. There seems to be a bi-directional relationship between migraine and depression, with each disorder increasing the risk of the other [11, 12]. Photophobia also was reported in patients with depression [13], but the nature of this relationship has not been rigorously studied. To the best of our knowledge, the relationship between interictal photophobia associated with migraine and depression and anxiety has never been investigated. In this observational pilot study we wished to better understand the relationships between interictal photophobia, depression and anxiety. We hypothesized that individuals with interictal photophobia and episodic migraine would have more anxiety and depression than those without interictal photophobia. We approached this question by determining the prevalence of symptoms of depression and anxiety in migraine patients with interictal photophobia, migraine patients without interictal photophobia and a control group.

## Methods

University of Utah IRB approved this study (IRB # 35510). All authors had full access to all study data. Potential participants were recruited from the clinics of three investigators (BJK, JEAW, KBD). Recruitment and collection of study data took place between July 2009 and June 2011. After sending potential participants an introductory letter, one of the investigators (SML, JEF) contacted each participant by phone to discuss the study. Subsequently, participants signed the informed consent document during a clinic visit, and before completing the study questionnaires. Over a 3 month time we recruited a total of 48 subjects: 16 participants with episodic migraine and interictal photophobia, 16 age-and gender-matched migraine subjects without interictal photophobia, and 16 age- and gender-matched subjects with neither migraine nor photophobia. All migraine subjects met the International Classification of Headache Disorders (ICHD) (second edition) criteria for episodic migraine with or without aura (i.e., headaches less than 15 days/month) [14]. None were wearing sunglasses indoors, or spectacles that were specifically tinted for migraine and/or photophobia. None were being treated for interictal photophobia. We excluded potential subjects with chronic migraine, medication overuse headache, or chronic tension type headache. We did not specifically include or exclude subjects with a known history of depression or anxiety.

We asked each migraine subject whether she/he had light sensitivity between migraine attacks. If a subject answered affirmatively, the subject was assigned to the “migraine with interictal photophobia” group. If they answered negatively, the subject was placed in the “migraine without interictal photophobia” group. Control subjects were recruited from family members and others who accompanied study subjects, and from clinic patients without episodic migraine. All control subjects were matched to the other two groups according to age and gender.

**Fig. 1:**
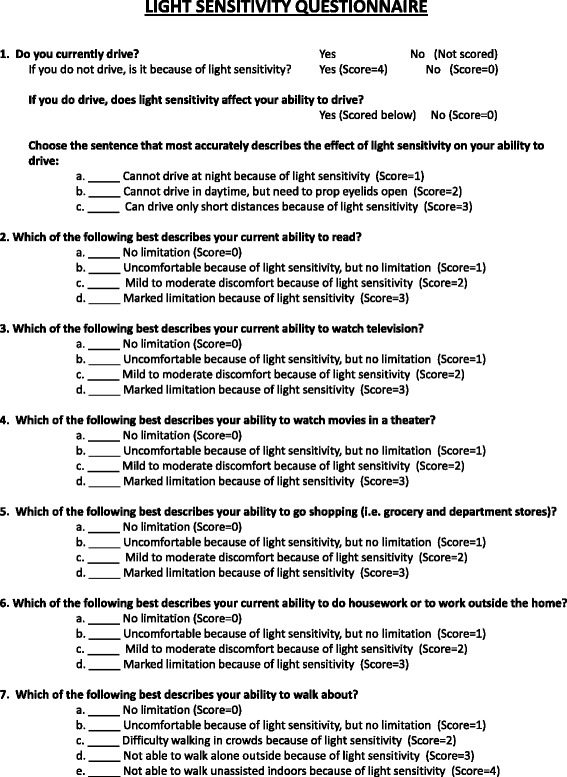
Photophobia questionnaire. This questionnaire was developed based on previous work by our group and by other groups for the assessment of photophobia in migraine and benign essential blepharospasm, two neurologic disorders that are strongly associated with photophobia. We developed this version to quantify the severity of light sensitivity and assess its effect on some activities of daily living

To assess the presence and impact of photophobia, subjects completed a photophobia questionnaire (Fig. 1). We developed this questionnaire from questionnaires that have been used to assess photophobia in previous investigations [15, 16]. During the same session, subjects also completed the Beck Depression Inventory II (BDI-II) and the Beck Anxiety Inventory (BAI), validated methods to assess for symptoms of depression and anxiety, respectively [17–20]. The BDI-II consists of 21 questions, each with a score between 0 and 3. The minimum score is 0 and the maximum score is 63. Scores between 0 and 13 are scored as “minimal” depression, scores between 14 and 19 are scored as “mild” depression, scores between 20 and 28 are scored as “moderate” depression and scores 29 and above are considered to be consistent with “severe” depression. The BAI also consists of 21 questions, each with a score between 0 and 3. Scores between 0 and 21 are interpreted as low anxiety, scores between 22 and 35 are consistent with moderate anxiety, and scores greater than or equal to 36 are interpreted as evidence of severe anxiety.

### Statistical analysis

We used Microsoft Excel 2010 and R 3.2.2 for the analyses. Pearson chi-square analyses and Wilcoxon rank sum tests were used to compare the cohorts. A *P* value of < 0.05 was considered significant. All tests were two sided. The sample size was determined by the number of patients that were seen for photophobia or light sensitivity while the study was open for enrollment.

## Results

The mean age of subjects was 51.5 years and all three groups consisted of 2 men and 14 women. Using the photophobia questionnaire, subjects with interictal photophobia obtained a mean score of 7.9 (standard deviation [S.D.] = 3.9), migraine subjects without interictal photophobia obtained a mean score of 0.9 (S.D. = 0.5) and control subjects had a mean score of 1.0 (S.D. = 0.7). The differences between the migraine with interictal photophobia group and the migraine without interictal photophobia group (*P* < 0.001) and the control group (*P* < 0.001) were both statistically significant. The photophobia scores for the migraine without interictal photophobia group and the control group were similar (*P* = 0.756)

**Fig. 2:**
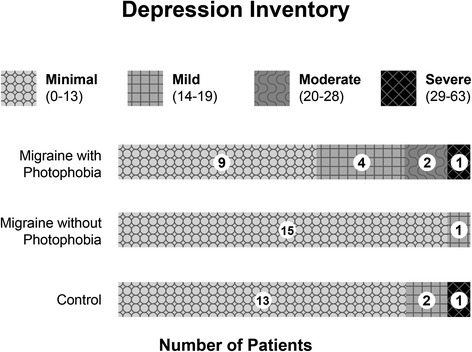
Depression Inventory scores in migraine subjects with interictal photophobia, migraine subjects without interictal photophobia and a control group. Subjects with interictal photophobia were more likely to have depression inventory scores consistent with mild, moderate or severe depression compared to the other two groups

The averaged depression inventory total score (i.e., the sum of the scores of all the subsets) was higher for patients with migraine with interictal photophobia (mean = 13; S.D. = 10) than for both migraine patients without interictal photophobia (mean = 6; S.D. = 4.7; *P* = 0.021) and controls (mean = 6; S.D. = 8.8; *P* = 0.0245). Seven interictal photophobia subjects had depression inventory total scores that indicated symptoms of depression that were mild, moderate or severe. One migraine subject had mild symptoms of depression. Two control subjects had mild symptoms of depression and one control subject had symptoms of severe depression (Fig. 2).

**Fig. 3:**
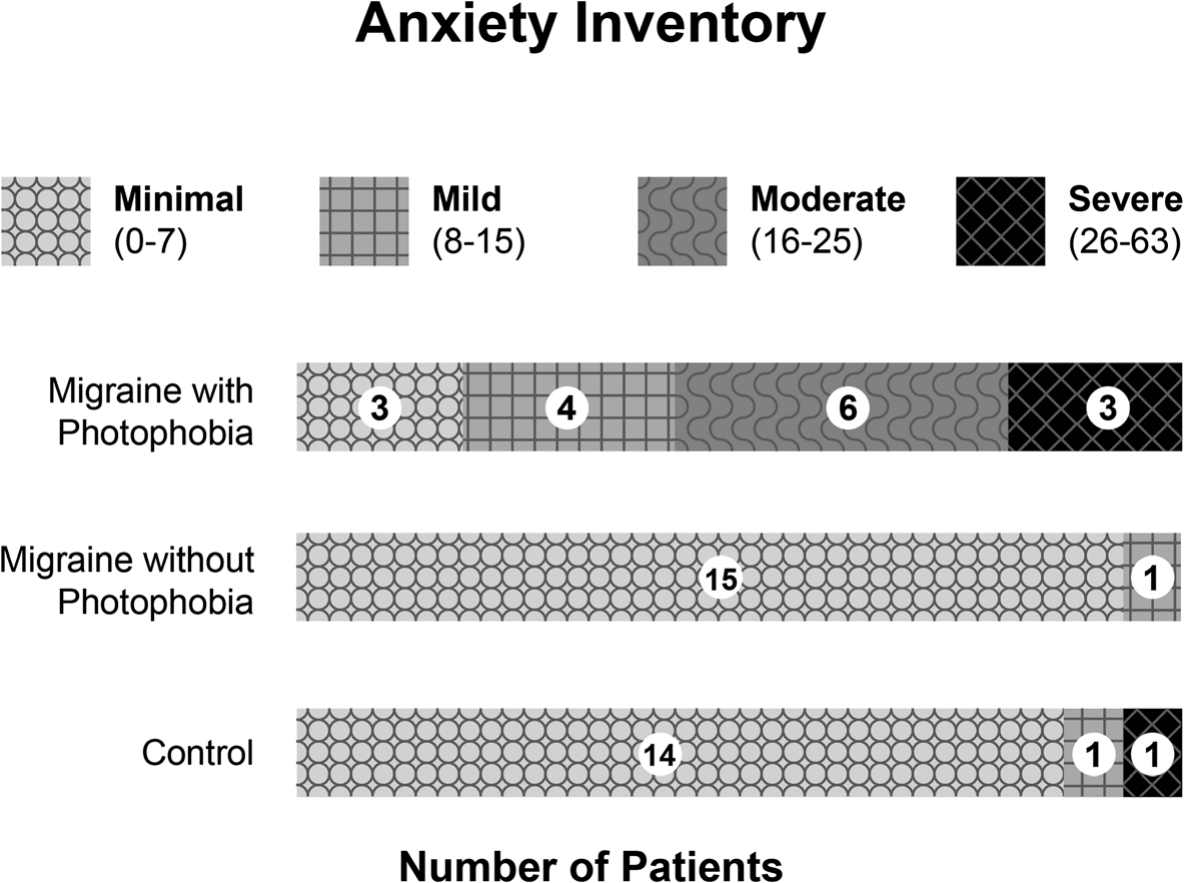
Anxiety Inventory scores in migraine subjects with interictal photophobia, migraine subjects without interictal photophobia, and a control group. Subjects with interictal photophobia were far more likely to have anxiety inventory scores consistent with mild, moderate or severe anxiety compared to the other two groups

The averaged anxiety inventory total score was significantly higher for patients with migraine and interictal photophobia (mean = 16; S.D. = 9.8) than for both migraine patients without interictal photophobia (mean = 4; S.D. = 3.4; *P* < 0.001 compared to interictal photophobia) and controls (mean = 5; S.D. = 6.9; *P* < 0.001 compared to interictal photophobia). Figure 3 illustrates the marked differences between interictal photophobia and the other two groups. Thirteen migraine subjects with interictal photophobia had symptoms of anxiety that were mild, moderate or severe. One migraine subject without interictal photophobia had symptoms of mild anxiety; one control subject had symptoms of mild anxiety and one control subject had symptoms of severe anxiety.

In the process of analyzing the data from the depression inventory, we noticed a marked difference in the prevalence of self-reported sleep problems among the three groups. Twelve of the 16 migraine with interictal photophobia subjects (75 %) reported changes in sleeping patterns (hypersomnia or insomnia) compared to seven control subjects (43.75 %) and six migraine without interictal photophobia subjects (37.5 %). This difference was statistically significant (*P* = 0.012).

## Discussion

Subjects who were assigned to the migraine with interictal photophobia group scored higher on the photophobia questionnaire than the other two groups. This result indicates that migraine subjects with interictal photophobia were appropriately assigned to this group based on the clinical interview. Subjects with migraine without interictal photophobia had photophobia questionnaire scores similar to the control group, also indicating that the migraine subjects without photophobia were assigned to the appropriate group based on the clinical interview.

Previous studies have demonstrated an association between migraine, anxiety and depression [5–10]. Researchers have proposed that the relationship between migraine, depression and anxiety is bi-directional, that is that they both influence each other [11, 12]. Previous work has also demonstrated that migraine patients are more light sensitive (“photophobic”) than control subjects [15, 21–23], in some cases even between attacks [3]. Here we have demonstrated that symptoms of depression and anxiety are more prevalent among patients with migraine with interictal photophobia. We did not specifically include or exclude subjects with a known history of depression or anxiety. Photophobia may be a marker of more severe migraine (although in this study, not chronic migraine). The mean age of our subjects was 51 years, perhaps an indication of inter-ictal photophobia acquired over years of migraine. Interictal photophobia could represent central sensitization due to a lengthy migraine history.

Our study suggests that the relationship between migraine with interictal photophobia, anxiety and depression may indeed be multidirectional, with each of these entities influencing the other. To our knowledge, there have been no studies of anxiety or depression in other conditions characterized by interictal photophobia (such as iritis). One study of subjects with tinnitus, another distressing subjective neuro-sensory symptom, indicated that report of poor quality of life was mediated by anxiety and depression [24].

Our final observation, that subjects with migraine with interictal photophobia are more likely to report sleep problems was marked, but perhaps not unexpected, given the concomitant symptoms of depression and anxiety. Animal models also suggest a connection between emotional brain centers and light sensitivity. Delwig et al. found that in neonatal mice, which have no formed vision, light evoked vocalizations similar to vocalizations interpreted as anxiety of being isolated from the mother, and patterns of neural activation of the central amygdala and the posterior thalamic group, brain regions that are involved in pain processing and photophobia [25, 26]. The correlation between sleep disorders and migraine with interictal photophobia may be related to the neural circuits that underlie photophobia [27]. Circadian rhythms are entrained by the intrinsically photosensitive retinal ganglion cells of the eye [28]. These same cells appear to be the origin of a neural pathway that mediates the exacerbation of migraine pain by light [26].

We propose that patients who have migraine and interictal photophobia may harbor an abnormality in these neural circuits that results in both photophobia and disturbances of the sleep cycle. If the link between symptoms of anxiety and depression and migraine with interictal photophobia is confirmed by larger studies, this may offer avenues for treatment. There is good evidence that treating migraine improves depression and anxiety and that treating depression and anxiety favorably influences the treatment of migraine [12, 29–31]. What remains to be shown is if treatment of photophobia improves depression and/or anxiety?

Our study group was small and recruited from a tertiary care center, and these limitations should be taken into consideration when extrapolating our results to other patients with migraine and interictal photophobia. Nonetheless, we feel that the results we observed in this small sample merit additional investigation into the interaction between migraine, aura, photophobia, depression and anxiety, particularly by including a larger sample size from primary care settings that serve a variety of ethnic populations. Previous studies have documented differences in the clinical course of those who are members of ethnic or racial groups other than the Caucasian population we encounter in our clinics [32–38]. The photophobia questionnaire we used for this study will provide a valuable tool for further definition and assessment of these potentially important differences. We look forward to such investigations in the future, and we are optimistic that this line of research will improve our ability to care for these patients.

## Conclusions

Most migraine specialists are aware of the increased prevalence of depression and anxiety in some of their patients. Our results indicate that if a migraine patient reports light sensitivity between attacks, they are at higher risk for these two mental health disorders. Physicians should consider having such patients evaluated and if necessary, treated for depression and anxiety. Because of the higher prevalence of self-reported sleep problems in this group and because of the adverse influence of poor sleep on migraine, physicians may also wish to consider inquiring about sleep problems and then recommend further evaluation or treatment in this group of patients.
